# STAT1 facilitates oestrogen receptor α transcription and stimulates breast cancer cell proliferation

**DOI:** 10.1111/jcmm.13882

**Published:** 2018-10-17

**Authors:** Yingxiang Hou, Xin Li, Qianhua Li, Juntao Xu, Huijie Yang, Min Xue, Gang Niu, Shu Zhuo, Kun Mu, Gaosong Wu, Xiumin Li, Hui Wang, Jian Zhu, Ting Zhuang

**Affiliations:** ^1^ Laboratory of Molecular Oncology Henan Collaborative Innovation Center of Molecular Diagnosis and Laboratory Medicine School of Laboratory Medicine Xinxiang Medical University Xinxiang Henan China; ^2^ Henan Key Laboratory of Immunology and Targeted Drugs Xinxiang Medical University Xinxiang Henan China; ^3^ Institute of Lung and Molecular Therapy (ILMT) Xinxiang Medical University Xinxiang Henan China; ^4^ Department of Pathology Shandong University School of Medicine Jinan Shandong China; ^5^ Rhil Rivers Technology (Beijing) Ltd Beijing China; ^6^ Department of Cancer Genomics LemonData Biotech (Shenzhen) Shenzhen China; ^7^ Department of Molecular Biology UT Southwestern Medical Center Dallas Texas; ^8^ Department of Thyroid and Breast Surgery Zhongnan Hospital Wuhan University Wuhan Hubei China; ^9^ Center for Cancer Research Xinxiang Medical University Xinxiang Henan China

**Keywords:** breast cancer, ERα, STAT1, transcription

## Abstract

Oestrogen receptor α (ERα) is overexpressed in two‐thirds of all breast cancer cases and is involved in breast cancer development and progression. Although ERα ‐positive breast cancer can be effectively treated by endocrine therapy, endocrine resistance is an urgent clinical problem. Thus, further understanding of the underlying mechanisms involved in ERα signalling is critical in dealing with endocrine resistance in patients with breast cancer. In the present study, unbiased RNA sequence analysis was conducted between the MCF‐7 and MCF‐7 tamoxifen‐resistant (LCC2) cell lines in order to identify differentially expressed genes. The whole transcriptomic data indicated that the JAK‐STAT pathway is markedly up‐regulated, particularly the ISGF3 complex. As the critical effectors, STAT1 and IRF9 were up‐regulated 5‐ and 20‐fold, respectively, in LCC2 cells. The biological experiments indicated that STAT1 is important for ERα signalling. Depletion of STAT1 or inhibition of STAT1 function significantly decreased levels of ERα protein, ERα ‐target gene expression and cell proliferation in both the MCF‐7 and LCC2 cell lines. Chromatin immunoprecipitation revealed that ERα transcription is associated with STAT1 recruitment to the ERα promoter region, suggesting that transcriptional regulation is one mechanism by which STAT1 regulates ERα mRNA levels and ERα signalling in breast cancer cells. The present study reveals a possible endocrine‐resistant mechanism by which STAT1 modulates ERα signalling and confers tamoxifen resistance. Targeting of STAT1 is a potential treatment strategy for endocrine‐resistant breast cancers.

## 1 INTRODUCTION

Breast cancer is the most frequently diagnosed type of cancer in women worldwide.^1^ A total of 60%‐70% of breast cancer cases are ERα positive, which can be well‐controlled by ERα selective antagonists such as tamoxifen.^2^ Tamoxifen has a similar structure as E2; however, it has an extra chain that interferes with the conformational change of ERα protein into the active form.^3^ Despite the effectiveness of tamoxifen treatment, a significant percentage of tumours with ERα expression develops endocrine resistance.^4^

A number of different mechanisms have been shown to account for tamoxifen resistance. For example, ERα acquires constitutively active mutations at the ligand binding domain: Y537S and D538G. These ERα mutant forms recruit the co‐activators in the absence of oestrogen, while the affinity for ERα antagonists is also decreased.^5^ ERα protein activity is also regulated by various post‐translational modifications, such as phosphorylation and ubiquitination.^6^, ^7^, ^8^ Several studies have shown that certain modifications at the ERα hinge domain enhance ERα transcriptional activity and confer tamoxifen resistance.^7^, ^8^ In addition to the active biological events in ERα signalling, a number of signalling pathways crosstalk with ERα via several effects. For example, numerous growth factor signalling kinases regulate ERα phosphorylation, including MAPK, RAS, AKT and PKA,^9^, ^10^, ^11^ which subsequently enhance ERα stability or/and transcriptional activity and renders cells less sensitive to tamoxifen.

Although a number of possible and confirmed mechanisms have been shown to explain endocrine resistance in breast cancer, how endocrine resistance is generated in breast cancer remains unclear. The LCC2 cell line, which was selected from the MCF‐7 cell line for tamoxifen resistance in oophorectomized nude mice, is widely used as an evolutionary model for tamoxifen‐resistant breast cancer.^12^ This model was utilized in the present study in order to perform unbiased RNA sequencing. By comparing the transcriptomic profiles of the MCF‐7 and LCC2 cell lines, JAK‐STAT signalling was observed to be expressed at higher levels in LCC2 cells. As the main effectors of JAK‐STAT signalling, STAT1 and IRF9 were markedly up‐regulated in the LCC2 cells. STAT was shown to be elevated in breast cancer tumours, while its expression levels correlated with poor endocrine treatment outcome. The present study identified the involvement of STAT1 in facilitating ERα transcription in breast cancer cells.

## 2 MATERIALS AND METHODS

### 2.1 Cell culture

MCF‐7 and LCC2 cell lines were used in our previous study.^13^ The cells were cultured in DMEM (Invitrogen, Carlsbad, CA) supplemented with 10% foetal bovine serum and 1% penicillin/streptomycin (Invitrogen) at 37°C in a humidified atmosphere of 5% CO_2_ in air.

### 2.2 siRNA transfection

Cells were transfected with 50 nM siRNA. STAT1 siRNA sequences were as follows: no. 1, 5′‐CUCAUUCCGUGGACGAGGUdTdT‐3′; and no. 2, 5′‐CCUGAUUAAUGAUGAACUAdTdT‐3′. The control siRNA sequence was as follows: UUCUCCGAACGUGUCACGUTT. INTERFERin transfection reagent (Polyplus Transfection, Illkirch‐Graffenstaden, France; cat. no. 409‐10) was used according to the manufacturer's protocol. Plasmids were transfected using Lipofectamine 2000 (1662298; Invitrogen). The ERE‐TK‐luc reporter and the pRL‐TK control were described in a previous study.^7^

### 2.3 RNA extraction and qPCR analysis

RNeasy kits (Qiagen, Beijing, China) were used to extract total RNA. qPCR was performed as previously described.^14^ 36B4 was used as an internal control. Primer sequences for qPCR are shown in Table 1.

**Table 1:** Primer sequence information for qPCR and CHIP assay

Primer for Q‐PCR	
STAT1 F	gag ccg ccc ggt gat tg
STAT1 R	aca gca aat gaa act ttt ctg cg
GREB1 F	5‐cgt gtg gtg act gga gta gc‐3
GREB1 R	5‐acc tct tca aag cgt gtc gt‐3
PS2 F	5‐cat cga cgt ccc tcc aga aga g‐3
PS2 R	5‐ctc tgg gac taa tca ccg tgc tg‐3
PDZK1 F	5‐gcc agg ctc att cat caa aga‐3
PDZK1 R	5‐cct cta gcc cag cca agt ca‐3
ESR1 F	5‐gct acg aag tgg gaa tga tga aag‐3
ESR1 R	5‐tct ggc gct tgt gtt tca ac‐3
36B4 F	5‐ggc gac ctg gaa gtc caa ct‐3
36B4 R	5‐cca tca gca cca cag cct tc‐3
Primers for ChiP assay	
ESR1 promoter A F	5‐GGG ATC GCT CCA AAT CGA‐3
ESR1 promoter A R	5‐CTT GCC CTG ACA TTG GCT TAA‐3
ESR1 promoter B F	5‐TCA GAT GCC CCC TGT CAG TT‐3
ESR1 promoter B R	5‐CAG CCA GCC ACA GAC AGC TA‐3
ESR1 promoter E2 F	5‐CAG CCC AGC CAA CAT GGT‐3
ESR1 promoter E2 R	5‐GCC CGC CAG CTA ATT TTT TA‐3

### 2.4 Quantification of cell viability

MCF‐7 and LCC2 cells were transfected with siSTAT1 or siControl in 24‐well plates. After 24 hours, the cells were seeded into 96‐well plates. Cell numbers were determined using WST‐1 cell proliferation reagent as previously described.^15^

### 2.5 Western blotting

Cells were lysed with RIPA lysis buffer. Anti‐ ERα mouse (1D5, SC56833) was obtained from Santa Cruz Biotechnology (Shanghai, China). Anti‐ ERα rabbit (D8H8, cat. no. 8644), anti‐STAT1 (9172), phospho‐STAT1 (9167S) and anti‐actin (8H10D10) were acquired from Cell Signaling Technology (Pudong, Shanghai, China).

### 2.6 Luciferase assay

The luciferase activity was performed with the Dual‐Luciferase Reporter kit (Promega, Madison, WI, USA) . The ERE luciferase reporter was transfected together with the Renilla plasmid into the cells. Luciferase activity was measured after 24 hours.

### 2.7 Chromatin immunoprecipitation (ChIP) assay

ChIP assay was performed in our previous study. MCF‐7 cells were fixed for cross‐linking for 30 minutes. Subsequently, the cells were mixed with 0.1375 M glycine, washed with cold PBS/1 mmol L^−1^ PMSF and scratched into PBS/1 mmol L^−1^ PMSF for centrifugation. Cells were treated with SDS lysis buffer and sonicated for 10 minutes (30 seconds on/off). A ChIP assay kit (Millipore, 17‐295, Burlington, MA, USA) was used for the subsequent steps. The following antibodies were used in the ChIP experiments: anti‐STAT1 (cat. no. 9172) and anti‐ ERα rabbit (D8H8, cat. no. 8644). The primer sequences used for the ChIP assay are shown in Table 1.

### 2.8 RNA sequence analysis

The global gene expression analysis was based on the RNA sequencing platform from Beijing Genomic Institute. The RNA sequence data are deposited in the Gene Expression Omnibus database (accession number GSE118774). The analysis was performed for differentially expressed genes (*P* < 0.01 and fold change >2) using Ingenuity Pathway Analysis.

### 2.9 Statistics

Student's *t* test and Pearson correlation coefficient were used for comparisons. For multiple group comparison, ANOVA (Analysis of Variance) was used for comparisons. Tukey's test was used as the post‐hoc test after ANOVA text. *P* < 0.05 was considered to be significant.

## 3 RESULTS

### 3.1 The ISGF3 components STAT1 and IRF9 are up‐regulated in tamoxifen‐resistant cells and correlate with poor tamoxifen treatment outcome

**Figure 1:**
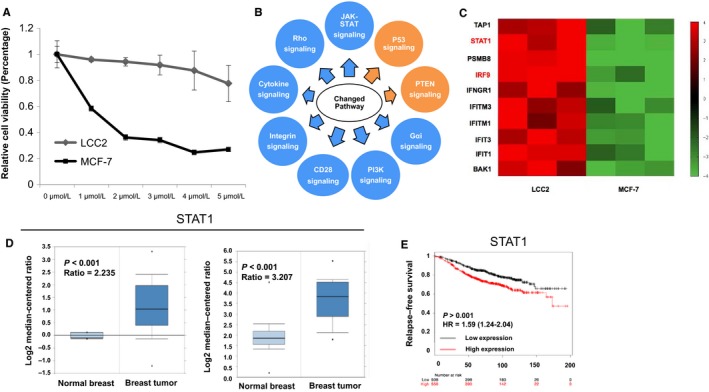
The ISGF3 components STAT1 and IRF9 are up‐regulated in tamoxifen‐resistant cells and correlate with poor tamoxifen treatment outcome. A, Comparison of tamoxifen sensitivity between MCF‐7 and LCC2 cells. MCF‐7 and LCC2 cells were treated with the indicated tamoxifen concentrations for 48 hours and the number of cells was quantified using a WST‐1 assay. Values are presented as the mean ± standard deviation for each concentration (n = 3). B, Top 10 signalling pathways significantly changed in LCC2 cells, compared to MCF‐7 cells. The pathway‐enrichment analysis was performed by the threshold *P* < 0.001 and fold change >2 in order to identify regulated genes. Each cell line was analyzed in triplicates. Blue arrows indicate activated signalling in LCC2 cells compared to MCF‐7 cells, while yellow arrows indicate inhibited signalling in LCC2 cells compared to MCF‐7 cells. C, The heat‐map graph shows the JAK‐STAT component genes, which are significantly increased in LCC2 cells compared to MCF‐7 cells. D, STAT1 gene expression is higher in breast tumours compared to normal breast tissue. E, STAT1 mRNA levels are correlated with poor endocrine treatment outcome in patients with breast cancer

**Figure 2:**
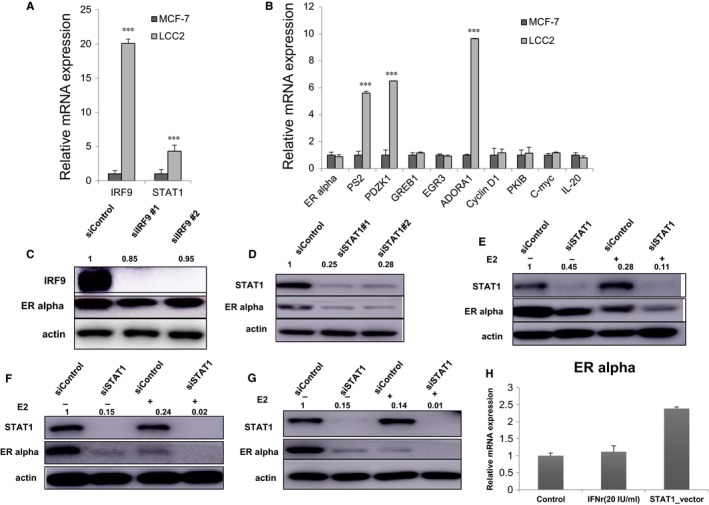
STAT1 depletion decreases ERα protein levels in breast cancer cells. A, IRF9 and STAT1 mRNA level is elevated in LCC2 cells compared with MCF‐7 cells. Total RNA was prepared and the expression of the endogenous IRF9 and STAT1 were determined by qPCR. ***P < 0.001 for target gene expression comparison. B, A subgroup of ERα target genes were increased in LCC2 cells compared with MCF‐7 cells. Total RNA was prepared and the expression of the endogenous ERα target genes were determined by qPCR. C, Effect of IRF9 depletion induced by two different siRNA oligos. MCF‐7 cells were transfected with siSTAT1 or siControl. After 48 hours, IRF9 and ERα protein levels were determined by western blot analysis. Actin was used as an internal control. The relative ERα protein levels were quantified with Image J. D, Effect of STAT1 depletion induced by two different siRNA oligos. MCF‐7 cells were transfected with siSTAT1 or siControl. After 48 h, STAT1 and ERα protein levels were determined by western blot analysis. Actin was used as an internal control. The relative ERα protein levels were quantified with Image J. E, Effect of STAT1 depletion on ERα protein levels under vehicle or E2 treatment conditions. MCF‐7 cells were transfected with siSTAT1 or siControl. After 48 hours, STAT1 and ERα protein levels were determined by western blot analysis. Actin was used as an internal control. The relative ERα protein levels were quantified with Image J. F, Effect of STAT1 depletion on ERα protein levels under vehicle or E2 treatment conditions. LCC2 cells were transfected with siSTAT1 or siControl. After 48 hours, STAT1 and ERα protein levels were determined by western blot analysis. Actin was used as an internal control. The relative ERα protein levels were quantified with Image J. G, Effect of STAT1 depletion on ERα protein levels under vehicle or E2 treatment conditions. T47D cells were transfected with siSTAT1 or siControl. After 48 h, STAT1 and ERα protein levels were determined by western blot analysis. Actin was used as an internal control. The relative ERα protein levels were quantified with Image J. H, STAT1 overexpression increases ERα mRNA level in MCF‐7 cells. MCF‐7 cells were seeded into 6‐well plates. After 24 hours, 2 μg STAT1 plasmids were transfected into MCF‐7 cells. After 48 hours, cells was harvested and ERα mRNA level was determined via QPCR

Firstly, the tamoxifen resistance of LCC2 cells compared to MCF‐7 cells was confirmed by measuring the IC50 of tamoxifen (Figure 1A). In order to compare the MCF‐7 and LCC2 cells in an unbiased way, the whole transcriptomic‐based RNA sequence was compared between these two cell lines. *P* < 0.001 was set as the significance threshold. In comparison with MCF‐7 cells, LCC2 cells activate a number of pathways, including the JAK‐STAT pathway, PI3K signalling and integrin signalling (Figure 1B and Table 2). In the JAK‐STAT pathway, several components were up‐regulated in the LCC2 cells, including STAT1 and IRF9 (Figure 1C). The qPCR and western blotting data showed that the STAT1 and IRF9 mRNA and protein levels were markedly increased (Figures 2A and 1C). The expression of several ER target genes was examined and it was markedly increased for a number of them, including PS2, PDZK1 and ADORA1 (Figure 2B). Previous studies have shown that ISGF3 functions as a critical transcription complex for JAK‐STAT activation. In the present study, STAT1/IRF9 expression in breast cancer samples was analysed. The Oncomine database showed that the mRNA expression levels of both STAT1 and IRF9 were elevated in breast tumours compared to normal breast tissues (https://www.oncomine.org/resource/login.html) (Figure 1D and Figure S1A). Publicly available survival data showed that both STAT1 and IRF9 correlated with poor endocrine treatment outcome (http://kmplot.com/analysis/) (Figure 1F and Figure S1B).

**Table 2:** Pathway analysis between MCF‐7 and LCC2 cells

Pathway Comparison between MCF‐7 and LCC2	*z*‐score
JAK‐STAT Signalling	3.32
Sphingosine‐1‐phosphate Signalling	2.40
UVA‐Induced MAPK Signalling	2.83
CXCR4 Signalling	2.56
IL‐8 Signalling	2.12
Rac Signalling	2.83
Chemokine Signalling	1.94
Cell Cycle: G2/M DNA Damage Checkpoint Regulation	1.90
Regulation of Actin‐based Motility by Rho	2.32
Remodelling of Epithelial Adherens Junctions	2.24
Actin Nucleation by ARP‐WASP Complex	1.90
Ephrin Receptor Signalling	2.84
Retinoic acid Mediated Apoptosis Signalling	3.00
Integrin Signalling	2.20
fMLP Signalling in Neutrophils	2.84
Signalling by Rho Family GTPases	2.50
Thrombin Signalling	2.04
Endothelin‐1 Signalling	2.29
Huntington's Disease Signalling	2.50
CD28 Signalling in T Helper Cells	2.14
Actin Cytoskeleton Signalling	2.29
Role of NFAT in Regulation of the Immune Response	2.36
Death Receptor Signalling	2.89
Gαi Signalling	2.53
RhoA Signalling	2.14
α‐Adrenergic Signalling	2.12
PI3K Signalling in B Lymphocytes	1.94
Dendritic Cell Maturation	2.52
Aryl Hydrocarbon Receptor Signalling	−1.00
RhoGDI Signalling	−2.40
p53 Signalling	−0.58
Oestrogen‐mediated S‐phase Entry	−1.34
Ephrin B Signalling	−0.82
Cyclins and Cell Cycle Regulation	−1.67
ErbB2‐ErbB3 Signalling	−0.71
PTEN Signalling	−0.58

### 3.2 STAT1 depletion decreases ERα mRNA and protein levels in breast cancer cells

**Figure 3:**
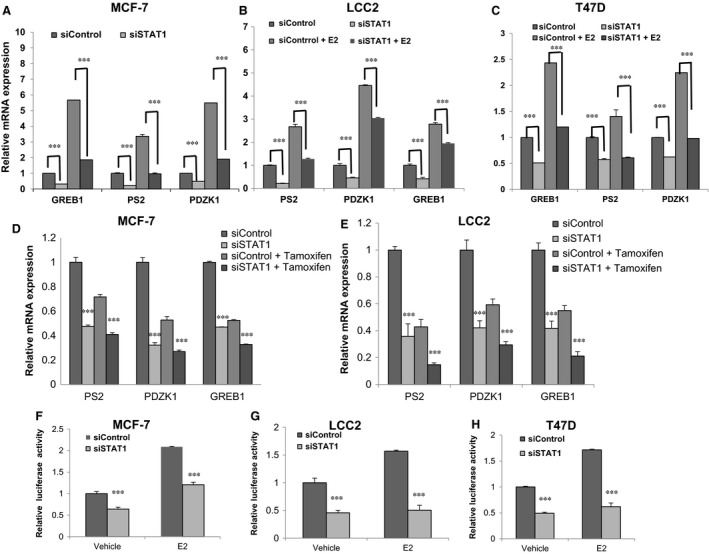
STAT1 depletion decreases ERα signalling in breast cancer cells. A, STAT1 depletion down‐regulates ERα target genes in MCF‐7 cells. MCF‐7 cells were transfected with siSTAT1 or siControl. After 48 hours, cells were cultured in phenol red‐free medium and treated with either ethanol or 10 nmol L^−1^ estradiol for 6 hours. Total RNA was prepared and the expression of the endogenous ERα target genes, PS2, GREB1 and PDZK1 were determined by qPCR. Results from three experiments are shown. ***P < 0.001 for target gene expression comparison. B, STAT1 depletion down‐regulates ERα target genes in LCC2 cells. LCC2 cells were transfected with siSTAT1 or siControl. After 48 h, cells were cultured in phenol red‐free medium and treated with either ethanol or 10 nmol L^−1^ estradiol for 6 h. Total RNA was prepared and the expression of the endogenous ERα target genes, PS2, GREB1 and PDZK1 were determined by qPCR. Results from three experiments are shown. ***P < 0.001 for target gene expression comparison. C, STAT1 depletion down‐regulates ERα target genes in T47D cells. T47D cells were transfected with siSTAT1 or siControl. After 48 hours, cells were cultured in phenol red‐free medium and treated with either ethanol or 10 nmol L^−1^ estradiol for 6 hours. Total RNA was prepared and the expression of the endogenous ERα target genes, PS2, GREB1 and PDZK1 were determined by qPCR. Results from three experiments are shown. ***P < 0.001 for target gene expression comparison. D, STAT1 depletion down‐regulates ERα target genes in tamoxifen‐treated condition in MCF‐7 cells. MCF‐7 cells were transfected with siSTAT1 or siControl. After 48 hours, cells were cultured in phenol red‐free medium and treated with either ethanol or 1 μmol L^−1^ tamoxifen for 6 h. Total RNA was prepared and the expression of the endogenous ERα target genes, PS2, GREB1 and PDZK1 were determined by qPCR. Results from three experiments are shown. ***P < 0.001 for target gene expression comparison. E, STAT1 depletion down‐regulates ERα target genes in tamoxifen‐treated condition in LCC2 cells. LCC2 cells were transfected with siSTAT1 or siControl. After 48 h, cells were cultured in phenol red‐free medium and treated with either ethanol or 1 μmol L^−1^ tamoxifen for 6 hours. Total RNA was prepared and the expression of the endogenous ERα target genes, PS2, GREB1 and PDZK1 were determined by qPCR. Results from three experiments are shown. ***P < 0.001 for target gene expression comparison. F, STAT1 depletion affects ERE‐luciferase activity in MCF‐7 cells. MCF‐7 cells were transfected with siSTAT1 or siControl together with a ERE luciferase reporter plasmid. Cells were treated with 10 nmol L^−1^ estradiol or vehicle. Luciferase activity was measured 48 hours after transfection. Results from three experiments are shown. ***P < 0.001 for luciferase activity comparison. G, STAT1 depletion affects ERE‐luciferase activity in LCC2 cells. LCC2 cells were transfected with siSTAT1 or siControl together with a ERE luciferase reporter plasmid. Cells were treated with 10 nmol L^−1^ estradiol or vehicle. Luciferase activity was measured 48 hours after transfection. Results from three experiments are shown. ***P < 0.001 for luciferase activity comparison. H, STAT1 depletion affects ERE‐luciferase activity in T47D cells. T47D cells were transfected with siSTAT1 or siControl together with a ERE luciferase reporter plasmid. Cells were treated with 10 nmol L^−1^ estradiol or vehicle. Luciferase activity was measured 48 hours after transfection. Results from three experiments are shown. ***P < 0.001 for luciferase activity comparison

The role of STAT1/IRF9 in oestrogen signalling was assessed. Depletion of STAT1 via two different siRNAs decreased ERα protein levels in the MCF‐7 cells (Figure 2D), but IRF9 depletion did not change ERα protein levels (Figure 2C). STAT1 was then depleted in both MCF‐7 and LCC2 cells in order to observe ERα protein levels in vehicle/E2 treatment conditions. STAT1 depletion decreased ERα protein levels in the vehicle/E2 treatment conditions in MCF‐7, LCC2 and T47D cells (Figure 2E‐G). In addition, STAT1 overexpression increased ERα levels (Figure 2H). qPCR showed that STAT1 depletion significantly decreases the expression levels of ERα target genes in MCF‐7, T47D and LCC2 cells, including those of PS2, GREB1 and PDZK1 (Figure 3A‐C). STAT1 depletion decreased ERα target gene expression levels in the MCF‐7 and LCC2 cells under both vehicle and tamoxifen treatment conditions (Figure 3D,E). By measuring ERE luciferase activity, STAT1 depletion was found to decrease ERα reporter gene activity under both vehicle and estradiol treatment in the MCF‐7, T47D and LCC2 cells (Figure 3F‐H).

### 3.3 STAT1 depletion inhibits breast cancer cell proliferation and sensitizes cells to the tamoxifen inhibition effect

**Figure 4:**
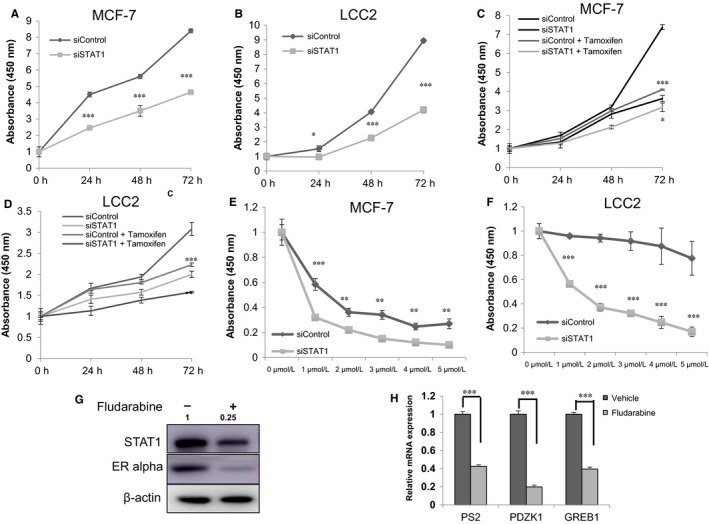
STAT1 depletion inhibits breast cancer cell proliferation and sensitizes cells to the tamoxifen inhibition effect. A, WST‐1 assay was used to determine the cellular metabolic activity at the indicated time points after transfection. MCF‐7 cells were transfected with siSTAT1 and siControl. After 24 hours, cells were seeded into 96‐well plates. These experiments were performed in triplicates. All values are presented as the mean ± standard deviation (n = 3, ***P < 0.001). B, WST‐1 assay was used to determine the cellular metabolic activity at the indicated time points after transfection. LCC2 cells were transfected with siSTAT1 and siControl. After 24 hours, cells were seeded into 96‐well plates. These experiments were performed in triplicates. All values are presented as the mean ± standard deviation (n = 3, ***P < 0.001). C, WST‐1 assay was used to determine the cellular metabolic activity at the indicated time points after transfection. MCF‐7 cells were transfected with siSTAT1 and siControl. After 24 hours, cells were seeded into 96‐well plates treated with vehicle or 1 μmol L^−1^ tamoxifen. These experiments were performed in triplicates. All values are presented as the mean ± standard deviation (n = 3, ***P < 0.001). D, WST‐1 assay was used to determine the cellular metabolic activity at the indicated time points after transfection. LCC2 cells were transfected with siSTAT1 and siControl. After 24 hours, cells were seeded into 96‐well plates treated with vehicle or 1 μmol L^−1^ tamoxifen. These experiments were performed in triplicates. All values are presented as the mean ± standard deviation (n = 3, ***P < 0.001). E, STAT1 depletion sensitized MCF‐7 cells to the tamoxifen inhibition effect. MCF‐7 cells were transfected with siSTAT1 and siControl. After 24 hours, cells were seeded into 96‐well plates. Cells were treated with the indicated concentration of tamoxifen for 48 hours and the number of cells was quantified using a WST‐1 assay. These experiments were performed in triplicates. All values are presented as the mean ± standard deviation (n = 3, ***P < 0.001). F, STAT1 depletion sensitized LCC2 cells to the tamoxifen inhibition effect. LCC2 cells were transfected with siSTAT1 and siControl. After 24 hours, cells were seeded into 96‐well plates. Cells were treated with the indicated concentration of tamoxifen for 48 hours and the number of cells was quantified using a WST‐1 assay. These experiments were performed in triplicates. All values are presented as the mean ± standard deviation (n = 3, ***P < 0.001). G, Pharmacological targeting of STAT1 by fludarabine inhibited ERα protein expression in breast cancer cells. MCF‐7 cells were treated with 5 μmol L^−1^ fludarabine for 24 hours. STAT1 and ERα protein levels were determined by western blot analysis. Actin was used as an internal control. H, Pharmacological targeting of STAT1 by fludarabine inhibited ERα target gene expression in breast cancer cells. MCF‐7 cells were treated by 5 μmol L^−1^ fludarabine for 24 hours. Total RNA was prepared and the expression of the endogenous ERα target genes, PS2, GREB1 and PDZK1 were determined by qPCR. Results from three experiments are shown. ***P < 0.001 for target gene expression comparison

In order to assess the effect of STAT1 on breast cancer cell proliferation, STAT1 was depleted in MCF‐7 and LCC2 cells. STAT1 depletion significantly decreased proliferation of the MCF‐7 and LCC2 cells under both vehicle and tamoxifen treatment conditions (Figure 4A‐D). STAT1 knockdown significantly sensitized both the MCF‐7 and LCC2 cells to the tamoxifen inhibition effect (Figure 4E,F). In order to confirm the effect of STAT1 on ERα signalling, fludarabine was used to specifically inhibit STAT1 expression. Figure 4G,H shows that 5 μM fludarabine effectively inhibits ERα protein levels and ERα target gene expression in breast cancer cells (Figure 4G,H).

### 3.4 Reduction of STAT1 levels reduces recruitment of STAT1 to the ERα promoter, which is a potential mechanism for ERα signalling regulation

**Figure 5:**
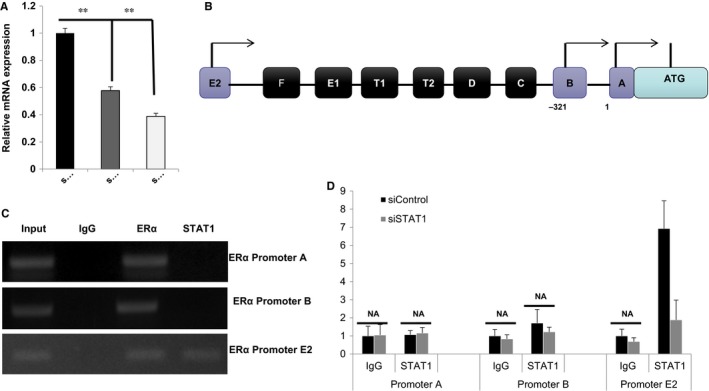
Reduction of STAT1 levels reduces recruitment of STAT1 to the ERα promoter, which is a potential mechanism for ERα signalling regulation. A, STAT1 depletion using two different siRNA oligos decreases ERα gene expression. MCF‐7 cells were transfected with siSTAT1 or siControl. After 48 hours, total RNA was prepared and the mRNA expression levels of endogenous ERα were determined using qPCR. Results from three experiments are shown. **P < 0.01 for gene expression comparison. B, Genomic organization of the ERα promoter structure of human ERα genes is shown, among which promoter A, promoter B and promoter E2 are used in MCF‐7 cells. C, ChIP assay showed that STAT1 is recruited to the ERα promoter E2. MCF‐7 cells were fixed for 30 minutes. Rabbit IgG was used as the negative control and ERα antibody was used as the positive control. The primer sequences are shown in Table 1. Enriched DNA fragments were then subjected to PCR and detected by DNA gel electrophoresis. D, ChIP assay showed that STAT1 depletion decreases STAT1 recruitment to ERα promoter regions. MCF‐7 cells were transfected with siSTAT1 or siControl for 48 hours. Cells were then fixed for 30 minutes. Rabbit IgG was used as the negative control. The primer sequences are shown in Table 1. The relative ERα promoter enrichment was measured by qPCR

**Figure 6:**
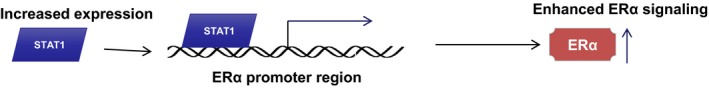
The illustrative model shows that STAT1 facilities ERα signalling activity possibly via promoting ERα transcription

ChIP assays were conducted in order to detect the possible association between STAT1 and ERα (data not shown). The IP assay using MCF‐7/LCC2 cells did not indicate the association between STAT1 and ERα. As ERα mRNA levels were also markedly decreased (Figure 5A), we have been suggested that STAT1 may regulate ERα at the transcriptional level. Seven promoters have been identified from ERα genes, while only promoters A, B and E2 are utilized for ERα expression in MCF‐7 cells (Figure 5B).^16^ ChIP assay was performed in order to detect STAT1 binding to ERα promoter regions. As ERα has been shown to bind to its own gene promoter regions, ERα antibody‐based ChIP was used as the positive control. The ChIP assay showed that STAT1 binds to the ERα promoter E2 but not to promoter A, while ERα binds to all three promoters (Figure 5C). Transfection with siRNA targeting STAT1 resulted in significantly decreased levels of binding at promoter E2 (Figure 5D). However, STAT1 activation via IFNr treatment does not increase STAT1 binding to ERα promoter regions (Figure S2A and S2B). Coupled with the data that show that STAT1 depletion significantly decreases ERα mRNA levels, it indicates that STAT1 binding to the ERα promoter region is a potential mechanism by which STAT1 facilitates ERα transcription and ERα signalling (Figure 6).

## 4 DISCUSSION

The JAK‐STAT signalling pathway consists of three main components: the cell surface receptor, JAK and STAT proteins.^17^ Among the transcription factors, STATs are the major effectors in regulation of target gene expression. For example, the ISGF3 complex, which consists of STAT1‐STAT2‐IRF9 proteins, binds to specific nucleotide sequences and is activated by interferon signalling.^18^ Previous studies showed that STAT1 plays both oncogenic and tumour‐suppression roles in various types of cancer, which may depend on the cancer cell background.^19^, ^20^ For example, STAT1 promoted oesophageal cancer invasion in the presence of p53 mutation,^21^ while STAT1 also induced cell cycle inhibition via interaction with cyclin D1 and CDKs.^22^ In breast cancer, STAT1 signalling correlates with poor endocrine treatment outcome, while the molecular mechanism is not clear.^23^ In the present study, STAT1 was found to be elevated in human breast cancer compared to normal breast tissues using a publicly available database. STAT1 is necessary to maintain ERα signalling in breast cancer cells, probably by regulating ERα gene expression. The present study offers a possible mechanism by which the JAK‐STAT pathway component STAT1 is involved in regulating oestrogen signalling activity and modulating tamoxifen sensitivity in breast cancer cells.

Since the development of endocrine therapy, tamoxifen has been used to treat patients with breast cancer for more than 40 years. This has resulted in a marked reduction in the mortality rate and remains one of the most effective treatments against breast cancer. Intensive research has been conducted in the past decades in order to investigate the underlying mechanism of endocrine resistance. In addition to the hyper‐activation of ERα signalling, either by a mutation for ERα constitutive activation or elevated ERα co‐activators,^5^, ^9^ the crosstalk between ERα signalling and other pathways also plays an important role in mediating tamoxifen resistance. A previous study found that MCF‐7 cells transfected with HER2 acquired tamoxifen resistance in xenograft mice models.^24^ Further studies have shown that ERα interacts with several other signalling pathways, including the HER2, EGFR and NFKB pathways.^25^, ^26^ In the present study, novel crosstalk between ERα signalling and the JAK‐STAT pathway was identified. As an important transcription factor, STAT1 may not only mediate JAK‐STAT activation, but also transactivate oestrogen signalling via modulation of ERα gene expression.

Along with the extensive studies of tamoxifen resistance in breast cancer, a number of tamoxifen‐resistant breast cancer cell lines have been derived, with the majority of which from tamoxifen‐sensitive MCF‐7 cells.^12^, ^27^, ^28^ Among them, LCC2 cells are the most frequently used tool for investigating the mechanism of tamoxifen resistance.^12^ In the present study, whole genomic expression profiles were compared between LCC2 and MCF‐7 cells. The pathway enrichment analysis showed higher expression levels of JAK‐STAT components, including STAT1. The data indicate that STAT1 is an important component in the regulation of ERα transcription in ERα ‐positive cancer cells. As modulation of ERα levels is one feasible approach to target oestrogen signalling and cell proliferation, STAT1 is a potential drug target for ERα ‐positive breast cancers.

## AUTHORS’ CONTRIBUTIONS

YXH, XL and QHL contributed to the manuscript writing. YXH, XL, QHL, HJY, MX, KM, GSW, XML and SZ contributed to the molecular and cellular biology experiments. JTX and GU contributed to the clinical data analysis and RNA sequence data analysis. TZ and HW contributed to scientific design, manuscript revising and the funding support for this study.

## AVAILABILITY OF DATA AND MATERIALS

Additional data and materials may be requested from the corresponding author on reasonable request.

## COMPETING INTERESTS

The authors declare that they have no competing interests.
