# Supplementary material for: STAT1 facilitates oestrogen receptor α transcription and stimulates breast cancer cell proliferation
Source: J Cell Mol Med. 2018 Oct 17;22(12):6077–86. doi: 10.1111/jcmm.13882 (PMC6237559; doi:10.1111/jcmm.13882)
Supplement: Supplementary file 1 [file JCMM-22-6077-s001.pptx]

## Slide 1
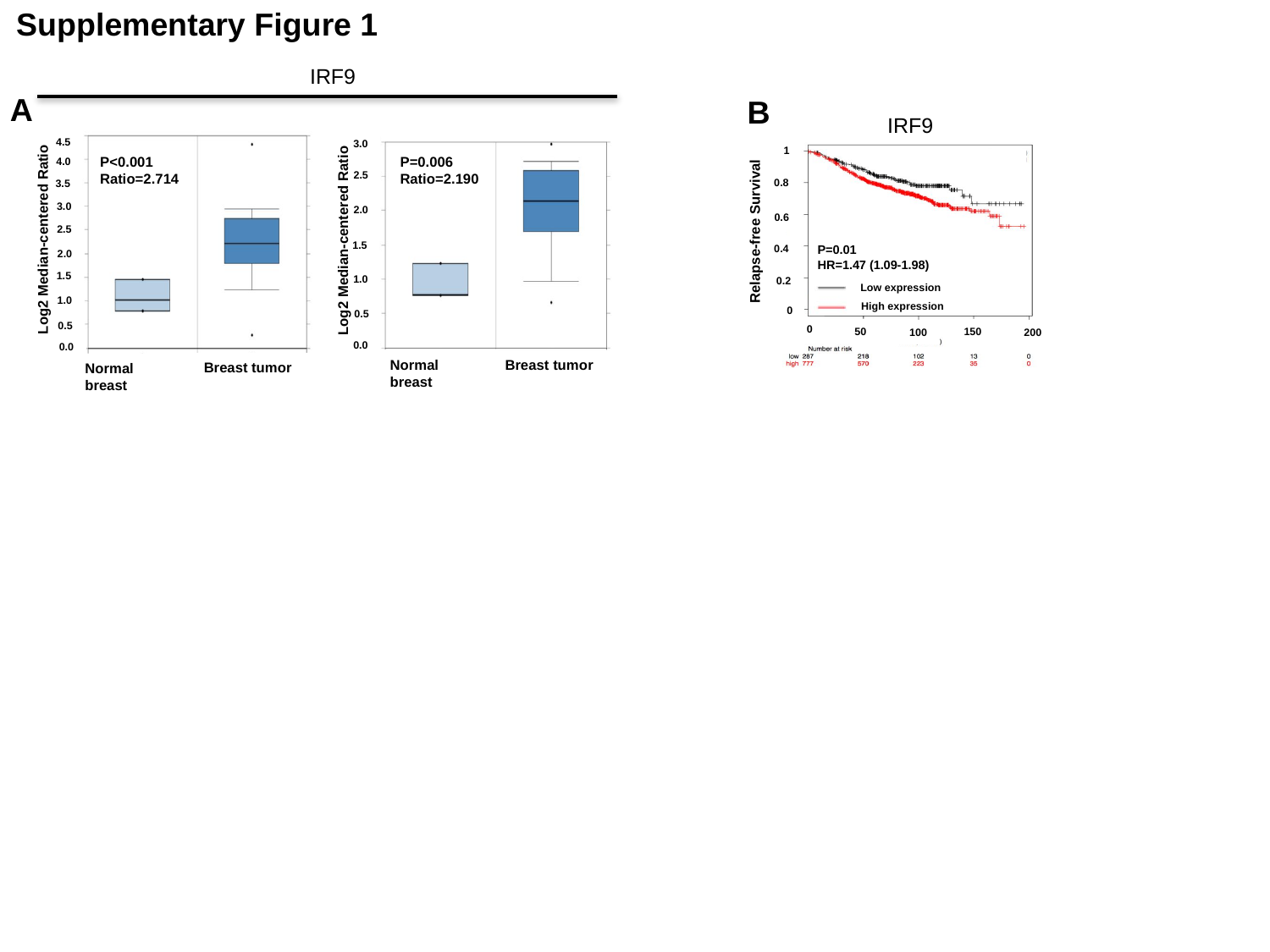

Supplementary Figure 1
IRF9
A
B
IRF9
4.5
P<0.001
Ratio=2.714
4.0
3.5
3.0
2.5
Log2 Median-centered Ratio
2.0
1.5
1.0
0.5
0.0
Breast tumor
Normal breast
3.0
1
P=0.006
Ratio=2.190
2.5
0.8
Relapse-free Survival
2.0
Relapse-free Survival
0.6
Log2 Median-centered Ratio
1.5
0.4
P=0.01
HR=1.47 (1.09-1.98)
1.0
0.2
Low expression
High expression
0
0.5
0
50
150
100
200
0.0
Normal breast
Breast tumor

## Slide 2
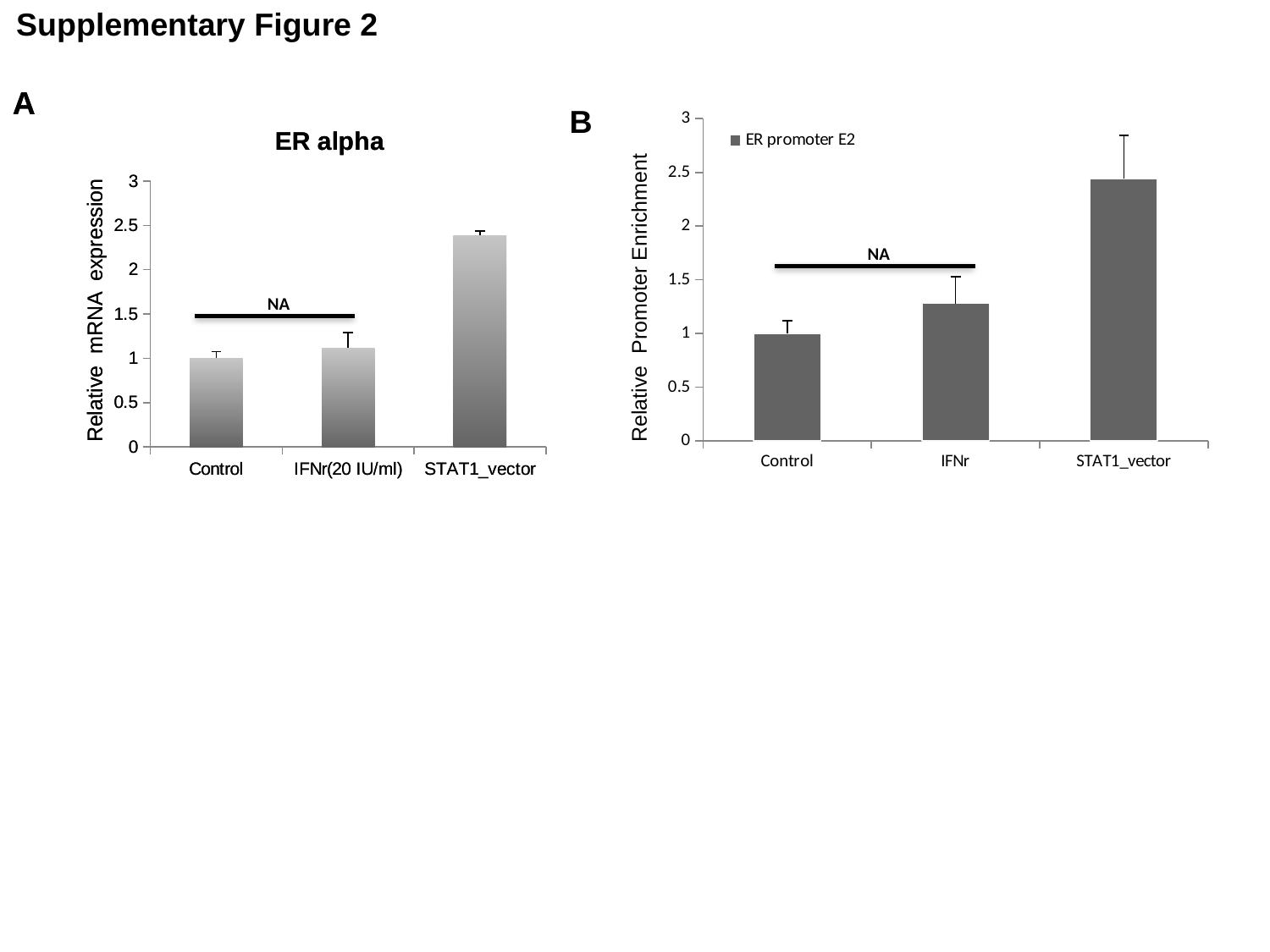

Supplementary Figure 2
A
A
B
### Chart
| Category | ER promoter E2 |
|---|---|
| Control | 1.0 |
| IFNr | 1.279076107224987 |
| STAT1_vector | 2.44050631281253 |
### Chart:
| Category | ER alpha |
|---|---|
| Control | 1.0 |
| IFNr(20 IU/ml) | 1.11596684640673 |
| STAT1_vector | 2.38570674611578 |
### Chart:
| Category | ER alpha |
|---|---|
| Control | 1.0 |
| IFNr(20 IU/ml) | 1.11596684640673 |
| STAT1_vector | 2.38570674611578 |NA
Relative mRNA expression
Relative mRNA expression
Relative Promoter Enrichment
NA
